# Supplementary material for: Interventions to improve racial and ethnic equity in critical care: A scoping review
Source: PLoS One. 2025 Nov 25;20(11):e0336922. doi: 10.1371/journal.pone.0336922 (PMC12646404; doi:10.1371/journal.pone.0336922)
Supplement: S1 Database — (DOCX) [file pone.0336922.s002.docx]

**Supplementary Material 2. Database Search Strategies**

**Focused Search**

Searches were limited to date of publication up to December 31, 2023. For each database, all fields, including title, abstract, subject headings, keywords, etc. were searched. All databases were last queried on May 31, 2024.

PubMed

*Search strategy:* ("race" OR "racial" OR "ethnic*") AND ("disparity" OR "disparities" OR "inequality" OR "inequalities" OR "inequity" OR "inequities") AND (("icu") OR ("critical care") OR ("intensive care") OR ("intensive care units") OR "critical illness" OR (ARDS) OR (respiratory failure) OR (ECMO) OR (sepsis)) AND ("quality improvement" OR "improvement" OR "improve" OR "reduction" OR "reduce" OR "intervention" OR “intervene”)

*Articles found:* 448

Web of Science

*Search strategy:* (ALL=(race) or ALL=(racial) or ALL=(ethnic*)) AND (ALL=(disparity) or ALL=(disparities) or ALL=(inequality) or ALL=(inequalities) or ALL=(inequity) or ALL=(inequities)) AND (ALL=(icu) or ALL=("critical care") or ALL=("intensive care") or ALL=("intensive care units") or ALL=("critical illness") or ALL=(ards) or ALL=(respiratory failure) or ALL=(ecmo) or All=(sepsis)) AND (ALL=("quality improvement") OR ALL=("improvement") OR ALL=("improve") OR ALL=("reduction") OR ALL=("reduce") OR ALL=("intervention") OR ALL=(“intervene”))

*Articles found:* 309

CINAHL

*Search strategy:* ("race" OR "racial" OR "ethnic*") AND ("disparity" OR "disparities" OR "inequality" OR "inequalities" OR "inequity" OR "inequities") AND (("icu") OR ("critical care") OR ("intensive care") OR ("intensive care units") OR "critical illness" OR (ARDS) OR (respiratory failure) OR (ECMO) OR (sepsis)) AND ("quality improvement" OR "improvement" OR "improve" OR "reduction" OR "reduce" OR "intervention" OR “intervene”)

*Articles found:* 108

Embase

*Search strategy:* ("race" OR "racial" OR "ethnic*") AND ("disparity" OR "disparities" OR "inequality" OR "inequalities" OR "inequity" OR "inequities") AND (("icu") OR ("critical care") OR ("intensive care") OR ("intensive care units") OR "critical illness" OR (ARDS) OR (respiratory failure) OR (ECMO) OR (sepsis)) AND ("quality improvement" OR "improvement" OR "improve" OR "reduction" OR "reduce" OR "intervention" OR “intervene”)

*Articles found:* 532

**Broad Search, 1^st^ Iteration**

Like the Focused Search, searches were limited to date of publication up to December 31, 2023. All databases were last queried on May 31, 2024.

PubMed

*Search strategy:* ("race" OR "racial" OR "ethnic*") AND ("disparity" OR "disparities" OR "inequality" OR "inequalities" OR "inequity" OR "inequities") AND (("icu") OR ("critical care") OR ("intensive care") OR ("intensive care units") OR "critical illness" OR (ARDS) OR (respiratory failure) OR (ECMO) OR (sepsis))

*Articles found:* 1,510

Web of Science

*Search strategy:* (ALL=(race) or ALL=(racial) or ALL=(ethnic*)) AND (ALL=(disparity) or ALL=(disparities) or ALL=(inequality) or ALL=(inequalities) or ALL=(inequity) or ALL=(inequities)) AND (ALL=(icu) or ALL=("critical care") or ALL=("intensive care") or ALL=("intensive care units") or ALL=("critical illness") or ALL=(ards) or ALL=(respiratory failure) or ALL=(ecmo) or All=(sepsis))

*Articles found:* 1,272

CINAHL

*Search strategy:* ("race" OR "racial" OR "ethnic*") AND ("disparity" OR "disparities" OR "inequality" OR "inequalities" OR "inequity" OR "inequities") AND (("icu") OR ("critical care") OR ("intensive care") OR ("intensive care units") OR "critical illness" OR (ARDS) OR (respiratory failure) OR (ECMO) OR (sepsis))

*Articles found:* 413

Embase

*Search strategy:* ("race" OR "racial" OR "ethnic*") AND ("disparity" OR "disparities" OR "inequality" OR "inequalities" OR "inequity" OR "inequities") AND (("icu") OR ("critical care") OR ("intensive care") OR ("intensive care units") OR "critical illness" OR (ARDS) OR (respiratory failure) OR (ECMO) OR (sepsis))

*Articles found:* 1,826

**Broad search, 2^nd^ Iteration**

Searches were again limited to date of publication up to December 31, 2023. All databases were last queried on April 22, 2025. The second iterative broad strategy explicitly queried subject headings in databases with subject heading fields and included additional keywords related to critical care. All four databases were re-queried to include the new keywords but only databases with subject heading fields were queried for subject headings (PubMed, Embase, and CINAHL).

PubMed

("race" OR "racial*" OR "ethnic*" OR "Black"[tw] OR "Blacks" OR "African American*" OR "Hispanic" OR "Latino" OR "Latina" OR "Latinx" OR "Asian*" OR "Pacific Islander*" OR "minorit*" OR "Racial Groups"[Mesh] OR "Minority Groups"[Mesh] OR "Minority Health"[Mesh] OR "Ethnicity"[Mesh] OR "Race Factors"[Mesh] OR "Racism"[Mesh]) AND ("disparity" OR "disparities" OR "inequality" OR "inequalities" OR "inequity" OR "inequities" OR "implicit bias" OR "racial bias" OR "ethnic bias" OR "Health Status Disparities"[Mesh] OR "Healthcare Disparities"[Mesh] OR "Health Equity"[Mesh] OR "Health Inequities"[Mesh:NoExp] OR "Bias, Implicit"[Mesh] OR "Racism"[Mesh] OR "Stereotyping"[Mesh] OR "Prejudice"[Mesh:NoExp]) AND (("icu") OR ("critical care") OR ("intensive care") OR ("intensive care units") OR "critical illness" OR (ARDS) OR (respiratory failure) OR (ECMO) OR (sepsis) OR "septic*" OR "critically ill" OR "respiratory distress" OR "extracorporeal membrane oxygen*" OR "extra corporeal membrane oxygen*" OR "Intensive Care Units"[Mesh] OR "Critical Care"[Mesh] OR "Critical Care Outcomes"[Mesh] OR "Critical Illness"[Mesh] OR "Sepsis"[Mesh] OR "Respiratory Distress Syndrome"[Mesh] OR "Extracorporeal Membrane Oxygenation"[Mesh])

NOT (“Geographic Locations”[Mesh] NOT “United States”[Mesh])

NOT (review[pt] OR editorial[pt] OR comment[pt] OR letter[pt])

*Articles found:* 1,506

CINAHL

("race" OR "racial*" OR "ethnic*" OR “Black” OR "Blacks" OR "African American*" OR "Hispanic*" OR "Latino*" OR "Latina*" OR "Latinx" OR "Asian*" OR "Pacific islander*" OR "minorit*" OR (MH "Minority Groups+") OR (MH "Ethnic Groups+") OR (MH "Race Factors") OR (MH "Racism+") OR (MH "Racialization")) AND ("disparity" OR "disparities" OR "inequality" OR "inequalities" OR "inequity" OR "inequities" OR "implicit bias" OR "racial bias" OR "ethnic bias" OR (MH "Equality") OR (MH "Implicit Bias") OR (MH "Healthcare Disparities") OR (MH "Health Status Disparities+") OR (MH "Health Inequities") OR (MH “Racial Equality”) OR (MH “Racism+”) OR (MH “Stereotyping”) OR (MH “Prejudice”)) AND ("icu" OR "critical care" OR "intensive care" OR "intensive care units" OR "critical illness" OR ARDS OR “respiratory failure” OR ECMO OR sepsis OR septic* OR "critically ill" OR "respiratory distress" OR "extracorporeal membrane oxygen*" OR "extra corporeal membrane oxygen*" OR (MH "Critical Care+") OR (MH "Critical Illness") OR (MH "Critically Ill Patients") OR (MH "Intensive Care Units+") OR (MH "Sepsis+") OR (MH "Respiratory Distress Syndrome, Acute") OR (MH "Respiratory Distress Syndrome, Newborn+") OR (MH "Extracorporeal Membrane Oxygenation"))

NOT ((MH "Geographic Locations+") NOT (MH "United States+"))

Limited to content from Academic Journals

*Articles found:* 505

Embase

((race OR racial* OR ethnic* OR Black OR Blacks OR African American* OR Hispanic* OR Latino* OR Latina* OR Latinx OR Asian* OR Pacific Islander* OR minorit*).ti,ab,hw OR exp ancestry group/ OR minority group/ OR minority health/ OR exp "ethnic or racial aspects"/ OR exp racism/) AND ((disparity OR disparities OR inequality OR inequalities OR inequity OR inequities OR implicit bias OR racial bias OR ethnic bias).ti,ab,hw OR health disparity/ OR health care disparity/ OR racial disparity/ OR health equity/ OR implicit bias/ OR stereotyping/ OR prejudice/) AND ((icu OR critical care OR intensive care OR intensive care units OR critical illness OR ARDS OR respiratory failure OR ECMO OR sepsis OR septic* OR critically ill OR respiratory distress OR extracorporeal membrane oxygen* OR extra corporeal membrane oxygen*).ti,ab,hw OR intensive care/ or icu triage/ or exp intensive care nursing/ or newborn intensive care/ OR exp intensive care unit/ OR critically ill patient/ OR critical illness/ OR exp respiratory distress syndrome/ OR exp sepsis/ OR exp extracorporeal oxygenation/)

NOT (exp geographic names/ not exp united states/)

NOT (books or chapter or editorial or erratum or note or "review").pt.

*Articles found:* 1,930

Web of Science

TS=((race OR racial* OR ethnic* OR Black OR Blacks OR “African American*” OR Hispanic* OR Latino* OR Latina* OR Latinx OR Asian* OR “Pacific Islander*” OR minorit*) AND (disparity OR disparities OR inequality OR inequalities OR inequity OR inequities OR “implicit bias” OR “racial bias” OR “ethnic bias” OR prejudice OR stereotyp*) AND (icu OR “critical care” OR “intensive care” OR “intensive care units” OR “critical illness” OR ARDS OR “respiratory failure” OR ECMO OR sepsis OR septic* OR “critically ill” OR “respiratory distress” OR “extracorporeal membrane oxygen*" OR “extra corporeal membrane oxygen*”))

Excluded reviews, editorial material, books, book chapters, notes

*Articles found:* 1,131

**Hand Search**

ClinicalTrials.gov, NIH RePORTER, MedRXiv, the Veterans Affairs (VA) Health Services Research and Development citations database, and journals and conference abstracts from the Society of Critical Care Medicine and the American Thoracic Society were each hand searched. These resources were each last searched on June 14, 2024.

*Terms used in hand search:* race, ethnicity, disparity, inequality, intervention, quality improvement

*Articles found:* 19
