# Supplementary material for: Interventions to improve racial and ethnic equity in critical care: A scoping review
Source: PLoS One. 2025 Nov 25;20(11):e0336922. doi: 10.1371/journal.pone.0336922 (PMC12646404; doi:10.1371/journal.pone.0336922)
Supplement: S1 Text — (DOCX) [file pone.0336922.s003.docx]

**Supplementary Material 3. Studies Excluded in Full-Text Review**

**Wrong Study Design**

Adams R, Henry K, Soleimani H, Rawat N, Saheed M, Chen E, et al. 1405: ASSESSING CLINICAL USE AND PERFORMANCE OF A MACHINE LEARNING SEPSIS ALERT FOR SEX AND RACIAL BIAS. Critical Care Medicine. 2022;50(1).

Allen, A., S. Mataraso, A. Siefkas, H. Burdick, G. Braden, R. P. Dellinger, A. McCoy, E. Pellegrini, J. Hoffman, A. Green-Saxena, G. Barnes, J. Calvert, and R. Das. 2020. 'A Racially Unbiased, Machine Learning Approach to Prediction of Mortality: Algorithm Development Study', *JMIR Public Health Surveill*, 6: e22400.

Allen, Jeremiah G., Eric S. Weiss, Christian A. Merlo, William A. Baumgartner, John V. Conte, and Ashish S. Shah. 2009. 'Impact of Donor-Recipient Race Matching on Survival After Lung Transplantation: Analysis of Over 11,000 Patients', *Journal of Heart and Lung Transplantation*, 28: 1063-71.

Alviar, C. L., R. A. D. U. Postelnicu, Pradhan D. R, Hena K. M, N. Chitkara, T. H. O. R. Milland, V. Mukherjee, A. M. I. T. Uppal, Goldberg R. I, M. Divita, F. Asef, K. A. H. Loon Wan, S. Vlahakis, M. Patel, M. A. R. O. S. A. R. I. O. Mertola, V. I. T. O. Stasolla, L. Bianco, Nunemacher K. M, V. Yunaev, Howe W. B, J. Cruz, S. Bernard, S. Bangalore, and Keller N. M. 2022. 'IMPROVING ACCESS TO ADVANCED CARDIORESPIRATORY THERAPIES FOR UNDERSERVED PATIENTS AND MINORITIES WITH A MULTIDISCIPLINARY EXTRACORPOREAL MEMBRANE OXYGENATION (ECMO) PROGRAM IN A LARGE PUBLIC HOSPITAL NETWORK', *Chest*, 162: A1111-A12.

Barnato, Amber E., Deepika Mohan, Julie Downs, Cindy L. Bryce, Derek C. Angus, and Robert M. Arnold. 2011. 'A randomized trial of the effect of patient race on physicians' intensive care unit and life-sustaining treatment decisions for an acutely unstable elder with end-stage cancer', *Critical Care Medicine*, 39: 1663-69.

Bhulani, Nizar, Arjun Gupta, M. Elizabeth Paulk, Kiauna Donnell, Valorie Harvey, Joan Cox, John Vernon Cox, Udit N. Verma, Aravind Sanjeevaiah, Naga Koteswari Cheedella, Leticia Khosama, Yull Edwin Arriaga, Samira K. Syed, Syed Mohammad Ali Kazmi, and Muhammad Shaalan Beg. 2018. 'Impact of palliative care consults on racial disparity in do-not resuscitation (DNR) orders at an urban safety net hospital', *Journal of Clinical Oncology*, 36.

Celedón, J. C., E. G. Burchard, D. Schraufnagel, C. Castillo-Salgado, M. Schenker, J. Balmes, E. Neptune, K. J. Cummings, F. Holguin, K. A. Riekert, J. P. Wisnivesky, J. G. N. Garcia, J. Roman, R. Kittles, V. E. Ortega, S. Redline, R. Mathias, A. Thomas, J. Samet, and J. G. Ford. 2017. 'An American Thoracic Society/National Heart, Lung, and Blood Institute Workshop Report: Addressing Respiratory Health Equality in the United States', *Ann Am Thorac Soc*, 14: 814-26.

Chen, I. Y., P. Szolovits, and M. Ghassemi. 2019. 'Can AI Help Reduce Disparities in General Medical and Mental Health Care?', *AMA J Ethics*, 21: E167-79.

Chima-Melton, C., T. E. Murphy, K. L. Araujo, and M. A. Pisani. 2016. 'The Impact of Race on Intensity of Care Provided to Older Adults in the Medical Intensive Care Unit', *J Racial Ethn Health Disparities*, 3: 365-72.

Chinman, Matthew, Eva N. Woodward, Geoffrey M. Curran, and Leslie R. M. Hausmann. 2017. 'Harnessing Implementation Science to Increase the Impact of Health Equity Research', *Medical Care*, 55.

Davila C, Cartagena L, Byrne-Martelli S, Bapat A, Stoltenberg M. Creating a Dedicated Palliative Care Team for ICU Spanish Speaking Patients in Response to COVID-19. Journal of Pain and Symptom Management. 2023;65(4):e315-e20.

Doshi, R., R. H. Aseltine, A. B. Sabina, and G. N. Graham. 2017. 'Interventions to Improve Management of Chronic Conditions Among Racial and Ethnic Minorities', *J Racial Ethn Health Disparities*, 4: 1033-41.

Galaviz, K. I., J. Y. Breland, M. Sanders, K. Breathett, A. Cerezo, O. Gil, J. M. Hollier, C. Marshall, J. D. Wilson, and U. R. Essien. 2020. 'Implementation Science to Address Health Disparities During the Coronavirus Pandemic', *Health Equity*, 4: 463-67.

Gerke, A. K., M. A. Judson, Y. C. Cozier, D. A. Culver, and L. L. Koth. 2017. 'Disease Burden and Variability in Sarcoidosis', *Ann Am Thorac Soc*, 14: S421-S28.

Harris, D. A., M. A. Pensa, C. A. Redlich, M. A. Pisani, and M. S. Rosenthal. 2016. 'Community-based Participatory Research Is Needed to Address Pulmonary Health Disparities', *Ann Am Thorac Soc*, 13: 1231-8.

Helfrich, C. D., C. W. Hartmann, T. J. Parikh, and D. H. Au. 2019. 'Promoting Health Equity through De-Implementation Research', *Ethn Dis*, 29: 93-96.

Iantorno, M., J. A. Panza, N. L. Cook, S. Jacobs, M. B. Ritchey, K. O'Callaghan, D. Caños, and H. A. Cooper. 2012. 'Gender- and race-based utilization and outcomes of pulmonary artery catheterization in the setting of full-time intensivist staffing', *Acute Card Care*, 14: 125-30.

Johnson, K., and M. Kuchibhatla. 2016. 'Did you get the DNR? Black-white differences in code status among older adults before and after inpatient palliative care consultation', *Journal of the American Geriatrics Society*, 64: S114.

Kalevor S, Uveges MK, Meyer EC. Using Everyday Ethics to Address Bias and Racism in Clinical Care. AACN Adv Crit Care. 2022;33(1):111-8.

Karanth, S., S. S. Rajan, F. L. Revere, and G. Sharma. 2019. 'Factors Affecting Racial Disparities in End-of-Life Care Costs Among Lung Cancer Patients: A SEER-Medicare-based Study', *Am J Clin Oncol*, 42: 143-53.

Landy, R., C. D. Young, M. Skarzynski, L. C. Cheung, C. D. Berg, M. P. Rivera, H. A. Robbins, A. K. Chaturvedi, and H. A. Katki. 2021. 'Using Prediction-Models to Reduce Persistent Racial/Ethnic Disparities in Draft 2020 USPSTF Lung-Cancer Screening Guidelines', *J Natl Cancer Inst*.

Lee, J. J., A. C. Long, J. R. Curtis, and R. A. Engelberg. 2016. 'The Influence of Race/Ethnicity and Education on Family Ratings of the Quality of Dying in the ICU', *J Pain Symptom Manage*, 51: 9-16.

Love, D. J., L. R. Nycum, T. S. Blackmon, A. J. Patefield, P. Bird, S. E. Stoker, R. A. Piersol, A. N. Koontz, and A. L. Collard. 2021. 'Simulation of "North Carolina Protocol for Allocating Scarce Inpatient Critical Care Resources in a Pandemic" in a Multi-hospital Health Care System', *N C Med J*, 82: 21-28.

McNulty, Moira, J. D. Smith, Juan Villamar, Inger Burnett-Zeigler, Wouter Vermeer, Nanette Benbow, Carlos Gallo, Uri Wilensky, Arthur Hjorth, Brian Mustanski, John Schneider, and C. Hendricks Brown. 2019. 'Implementation Research Methodologies for Achieving Scientific Equity and Health Equity', *Ethnicity & Disease*, 29: 83-92.

Napoles, A. M., and A. L. Stewart. 2018. 'Transcreation: an implementation science framework for community-engaged behavioral interventions to reduce health disparities', *BMC Health Serv Res*, 18: 710.

Rashdan, T., and S. Overstake. 2011. 'Bridging the gap: Culturally competent care for muslims', *Pediatric Critical Care Medicine*, 12: A135.

Rogowski, J. A., D. O. Staiger, and J. D. Horbar. 2004. 'Variations in the quality of care for very-low-birthweight infants: implications for policy', *Health Affairs*, 23: 88-97.

Sandoval, E., and D. W. Chang. 2016. 'Association Between Race and Case Fatality Rate in Hospitalizations for Sepsis', *J Racial Ethn Health Disparities*, 3: 625-34.

Sharma, Meesha, Wei Jiang, Muhammad A. Chaudhary, Anju Ranjit, Cheryl K. Zogg, Adil H. Haider, Andrew J. Schoenfeld, Peter Learn, and Tracey Koehlmoos. 2018. 'Universal Health Insurance and its association with long term outcomes in Pediatric Trauma Patients', *Injury*, 49: 75-81.

Siddique, S. M., C. V. Evans, M. Harhay, E. S. Johnson, J. Aysola, G. E. Weissman, N. K. Mull, E. Flores, H. Schmidt, K. Tipton, B. Leas, and J. S. Lin. 2023. 'Critical Appraisal for Racial and Ethnic Equity in Clinical Prediction Models Extension: Development of a Critical Appraisal Tool Extension to Assess Racial and Ethnic Equity-Related Risk of Bias for Clinical Prediction Models', *Health Equity*, 7: 773-81.

Snell-Rood, Claire, Elise Trott Jaramillo, Alison B. Hamilton, Sarah E. Raskin, Francesca M. Nicosia, and Cathleen Willging. 2021. 'Advancing health equity through a theoretically critical implementation science', *Translational Behavioral Medicine*, 11: 1617-25.

Soto, G. J., G. S. Martin, and M. N. Gong. 2013. 'Healthcare disparities in critical illness', *Crit Care Med*, 41: 2784-93.

Sterling, Madeline R., Sandra E. Echeverría, Yvonne Commodore-Mensah, Jessica Y. Breland, and Marcella Nunez-Smith. 2019. 'Health Equity and Implementation Science in Heart, Lung, Blood, and Sleep-Related Research', *Circulation: Cardiovascular Quality and Outcomes*, 12: e005586.

Thakur, N., S. Lovinsky-Desir, D. Appell, C. Bime, L. Castro, J. C. Celedón, J. Ferreira, M. George, Y. Mageto, Iii Ag Mainous, S. Pakhale, K. A. Riekert, J. Roman, E. Ruvalcaba, S. Sharma, P. Shete, J. P. Wisnivesky, and F. Holguin. 2021. 'Enhancing Recruitment and Retention of Minority Populations for Clinical Research in Pulmonary, Critical Care, and Sleep Medicine: An Official American Thoracic Society Research Statement', *Am J Respir Crit Care Med*, 204: e26-e50.

**Formative research (no intervention)**

Ashana, D. C., N. D'Arcangelo, P. K. Gazarian, A. Gupta, S. Perez, A. J. Reich, J. Tjia, S. D. Halpern, J. S. Weissman, and K. Ladin. 2022. '"Don't Talk to Them About Goals of Care": Understanding Disparities in Advance Care Planning', *J Gerontol A Biol Sci Med Sci*, 77: 339-46.

Barnato, Amber E. 2009. 'ICU Triage Decisions for Elders with End Stage Cancer: the Role of Patient Race'. Originally accessed on NIH RePORTER on 16 August 2021. <https://grantome.com/grant/NIH/R21-CA139264-01>.

Barwise, Amelia, Michael E. Wilson, Charat Thongprayoon, Rahul Kashyap, Brian W. Pickering, Vitaly Herasevich, and Ognjen Gajic. "Racial Disparities in End of Life Decision Making in the Intensive Care Unit: A Pilot Study." In *B105. IMPROVING PATIENT EXPERIENCE IN CRITICAL CARE*, A3777-A77.

Cartagena, D., J. M. McGrath, B. Reyna, L. A. Parker, and J. McInnis. 2022. 'Strategies to Improve Mother's Own Milk Expression in Black and Hispanic Mothers of Premature Infants', *Adv Neonatal Care*, 22: 59-68.

Dukkipati HS, Koch A, Misiewicz R, Grier K, Hepler B, Cox C, et al. Healthcare Providers as Cultural Guests of Patients and Their Loved Ones During the Dual Pandemic of COVID-19 and Racism: Establishing Trust in the ICU (Sci233). Journal of Pain and Symptom Management. 2023;65(5):e657-e8.

Enciso, J. M. 2020. 'Teaching culturally competent healthcare in neonatal-perinatal medicine', *Semin Perinatol*, 44: 151239.

Hilton, E. J., K. L. Goff, R. Sreedharan, N. Lunardi, M. Batakji, and D. S. Rosenberger. 2020. 'The Flaw of Medicine: Addressing Racial and Gender Disparities in Critical Care', *Anesthesiol Clin*, 38: 357-68.

Joseph, L., P. S. Chan, S. M. Bradley, Y. Zhou, G. Graham, P. G. Jones, M. Vaughan-Sarrazin, S. Girotra, and Investigators American Heart Association Get With the Guidelines-Resuscitation. 2017. 'Temporal Changes in the Racial Gap in Survival After In-Hospital Cardiac Arrest', *JAMA Cardiol*, 2: 976-84.

Kern-Goldberger, A. R., A. Friedman, L. Moroz, and C. Gyamfi-Bannerman. 2022. 'Racial Disparities in Maternal Critical Care: Are There Racial Differences in Level of Care?', *J Racial Ethn Health Disparities*, 9: 679-83.

Lake, E. T., D. Staiger, J. Horbar, M. J. Kenny, T. Patrick, and J. A. Rogowski. 2015. 'Disparities in perinatal quality outcomes for very low birth weight infants in neonatal intensive care', *Health Serv Res*, 50: 374-97.

Malhi M, Patel K, Danyalian A, Iguina MM, Lagrotta GA, Danckers M. IMPACT OF THE ICU-PAL INITIATIVE ON PALLIATIVE CARE CONSULTATION AND CLINICAL OUTCOMES IN AN INTENSIVE CARE UNIT SETTING. CHEST. 2023;164(4, Supplement):A5163-A4.

Meka, T, and K Tauber. 2022. 'Racial Disparities in Breastfeeding Rates for Preterm Infants: Opportunities for Change in an Ongoing Mother's Own Milk Quality Improvement Project', *Journal of Investigative Medicine*, 70: 1167-68.

Natale, J. E., L. A. Asaro, J. G. Joseph, C. Ulysse, J. Ascenzi, C. Bowens, D. Wypij, M. A. Q. Curley, and Restore Study Investigators. 2021. 'Association of Race and Ethnicity with Sedation Management in Pediatric Intensive Care', *Ann Am Thorac Soc*, 18: 93-102.

Parker, M. G., and S. S. Hwang. 2021. 'Quality improvement approaches to reduce racial/ethnic disparities in the neonatal intensive care unit', *Semin Perinatol*, 45: 151412.

Profit, J. 2018. 'A Dashboard of Racial/Ethnic Disparity in Care Provided by NICUs'. <https://reporter.nih.gov/search/8SGR6feETECYx8331cLFdg/project-details/9595395>.

Profit, J. 2019. 'Social Disparities in NICU Care'. <https://reporter.nih.gov/search/8SGR6feETECYx8331cLFdg/project-details/9627721>.

Profit, J., J. B. Gould, M. Bennett, B. A. Goldstein, D. Draper, C. S. Phibbs, and H. C. Lee. 2017. 'Racial/Ethnic Disparity in NICU Quality of Care Delivery', *Pediatrics*, 140.

Texeira-Poit, Stephanie. 2023. "Identifying and Mitigating Health Disparities Following a Neonatal Intensive Care Unit (NICU) Design Change from Open Bay to Single Family Rooms." In. NIH RePORTER.

Tyler, P. D., D. J. Stone, B. P. Geisler, S. McLennan, L. A. Celi, and B. Rush. 2018. 'Racial and Geographic Disparities in Interhospital ICU Transfers', *Crit Care Med*, 46: e76-e80.

Vujnic, Sanda, Daniel F. Dilling, and Lena Hatchett. 'Cultural Competence And Cross-Cultural Communication In The Medical Intensive Care Unit: Evaluation Of Patient Satisfaction With Care And Decision-Making Before An Educational Intervention Using The Family Satisfaction Questionnaire-24 (FS-ICU 24).' in, *C101. GAME CHANGER: PHYSICIAN PERFORMANCE IN PULMONARY AND CRITICAL CARE MEDICINE*.

**Wrong Outcomes**

Baggett, K. M., B. Davis, S. H. Landry, E. G. Feil, A. Whaley, A. Schnitz, and C. Leve. 2020. 'Understanding the Steps Toward Mobile Early Intervention for Mothers and Their Infants Exiting the Neonatal Intensive Care Unit: Descriptive Examination', *J Med Internet Res*, 22: e18519.

Banerjee, D., N. J. Nassikas, P. Singh, S. B. Andrea, A. Y. Zhang, Y. Aswad, N. Singh, S. R. Walsh, K. Cox-Flaherty, E. J. Carter, and K. M. Sharkey. 2022. 'Feasibility of an Antiracism Curriculum in an Academic Pulmonary, Critical Care, and Sleep Medicine Division', *ATS Sch*, 3: 433-48.

Brown-Madan, Y., A. Williams, and S. Langston. 2023. 'Addressing implicit bias and health disparities in a level IV NICU', *J Perinatol*, 43: 1494-96.

Chi, Amy, Rebecca Blanchard, and Elizabeth Bennett. 2013. 'Improving Cultural Competency In End-Of-Life Care In the Intensive Care Unit (ICU).' in, *D22. ADVANCING CRITICAL CARE THROUGH NEW APPROACHES AND PARADIGMS* (American Thoracic Society).

Chiu C, Martinez R, Masters D, Thornton K. Provider-Family Communication in the COVIDEra ICU, Improvements and Barriers: A Quality Improvement Project. Anesth Analg. 2021;132(5S Suppl 1):1-1072.

Freund, K. M., A. LeClair, N. Terrin, A. D. Hanchate, L. L. Price, A. Moreno-Koehler, J. Suzukida, S. Kher, E. Byhoff, and N. R. Kressin. 2019. 'Racial Differences in Insurance Stability After Health Insurance Reform', *Med Care*, 57: 256-61.

Haddad, D. N., K. L. Sandler, L. M. Henderson, M. P. Rivera, and M. C. Aldrich. 2020. 'Disparities in Lung Cancer Screening: A Review', *Ann Am Thorac Soc*, 17: 399-405.

Lawal O, Heyding D, Spiro P. Bridging the Gap: Improving Advance Directives and Shared Decision Making in the Medical ICU of an Urban Inner-City Hospital. Chest. 2011;140(4, Supplement):258A.

Mohamed, A., A. Shiari, D. Venkat, C. R. Jinjuvadia, A. O. Soubani, S. J. E. Lee, and A. Sankari. 2021. 'Can a New Blended Simulation Curriculum with Competency Assessment Ameliorate a Healthcare Disparity Gap in Interpreting Chest CT Imaging for Pulmonary and Critical Care Fellows?' in, *TP12. TP012 MEDICAL EDUCATION IN PULMONARY AND CRITICAL CARE MEDICINE* (American Thoracic Society).

Oguntebi, Abimbola Dairo, Emma M. Steinberg, Yana Vaks, and Tricia Tayama. 2022. 'A Different Type of Morbidity &amp; Mortality (M&amp;M) Conference: Advancing Racial and Health Equity via a Focused M&amp;M Series', *Pediatrics*, 149: 530-30.

Popovich, Kehllee, Janet Kloos, Jason Makii, Elisabeth Korosec, Elizabeth Hewett, Michael Dzigiel, stephanie Kubec, and Robert Snyder. 2022. '570: TRANSCENDING RACIAL AND SOCIAL DISPARITIES IN POST-ICU OUTCOMES', *Critical Care Medicine*, 50.

Yang MY, Kwak GH, Pollard T, Celi LA, Ghassemi M. Evaluating the Impact of Social Determinants on Health Prediction in the Intensive Care Unit. Proceedings of the 2023 AAAI/ACM Conference on AI, Ethics, and Society; Montréal, QC, Canada: Association for Computing Machinery; 2023. p. 333–50.

Zurbuchen, Rudi, Daniel Hekman, Ryan Tsuchida, Ciara Barclay-Buchanan, Marin Darsie, Brittney Bernardoni, and Joshua Glazer. 2023. '264: AN EQUITY DASHBOARD OF POTENTIALLY LIFE-SAVING INTERVENTIONS REVEALS UNDER-TRIAGE OF FEMALE PATIENTS', *Critical Care Medicine*, 51.

**Wrong Patient Population**

Helmers, Mark R., Cody Fowler, Peter Altshuler, Amit Iyengar, Jason J. Han, William Patrick, Edo Birati, and Pavan Atluri. 2020. 'Abstract 17085: The Modified US Heart Allocation System Improves Waitlist Mortality for Patients With Hypertrophic Cardiomyopathy', *Circulation*, 142: A17085-A85.

Howell, E. A., and J. Zeitlin. 2017. 'Improving hospital quality to reduce disparities in severe maternal morbidity and mortality', *Semin Perinatol*, 41: 266-72.

Jain, J., and L. Moroz. 2017. 'Strategies to reduce disparities in maternal morbidity and mortality: Patient and provider education', *Semin Perinatol*, 41: 323-28.

Keenan-Devlin, L. S., J. Y. Hughes-Jones, and A. E. B. Borders. 2021. 'Clinically integrated breastfeeding peer counseling and breastfeeding outcomes', *J Perinatol*, 41: 2095-103.

Schlenker, T., L. T. Dresang, M. Ndiaye, W. R. Buckingham, and J. W. Leavitt. 2012. 'The effect of prenatal support on birth outcomes in an urban midwestern county', *WMJ*, 111: 267-73.

Siva, Ajay, Ariba Hashmi, Connor Grabowski, Kathleen Henninger, Karen Zinnerstrom, Scott Gruarin, Regina Makdissi, Alysia Kwiatkowski, and Archana Mishra. 2023. 'IMPROVING CULTURAL COMPETENCY AND RESIDENT KNOWLEDGE USING CARDIAC ARREST SIMULATION', *Chest*, 164: A3848-A49.

**Wrong Setting**

Abshire, C., M. McDowell, A. H. Crockett, and N. L. Fleischer. 2019. 'The Impact of CenteringPregnancy Group Prenatal Care on Birth Outcomes in Medicaid Eligible Women', *J Womens Health (Larchmt)*, 28: 919-28.

Bazzoli, G. J., P. Carcaise-Edinboro, L. M. Sabik, P. Chandan, and S. Harpe. 2017. 'Integrated Case Management: Does It Reduce Health Service Disparities Across African American and White Medicaid Beneficiaries?', *Med Care Res Rev*, 74: 486-501.

Lau, B. D., A. H. Haider, M. B. Streiff, C. U. Lehmann, P. S. Kraus, D. B. Hobson, F. S. Kraenzlin, A. M. Zeidan, P. J. Pronovost, and E. R. Haut. 2015. 'Eliminating Health Care Disparities With Mandatory Clinical Decision Support: The Venous Thromboembolism (VTE) Example', *Med Care*, 53: 18-24.

Owodunni, O. P., E. R. Haut, D. L. Shaffer, D. B. Hobson, J. Wang, G. Yenokyan, P. S. Kraus, J. K. Aboagye, K. L. Florecki, K. L. W. Webster, C. G. Holzmueller, M. B. Streiff, and B. D. Lau. 2020. 'Using electronic health record system triggers to target delivery of a patient-centered intervention to improve venous thromboembolism prevention for hospitalized patients: Is there a differential effect by race?', *Plos One*, 15: e0227339.

Pruitt, D., K. Weber, and N. Ragina. 2021. 'Teaching end-of-life preparation to African Americans', *Palliat Support Care*, 19: 335-40.

Williams, N. J., R. Robbins, D. Rapoport, J. P. Allegrante, A. Cohall, G. Ogedgebe, and G. Jean-Louis. 2016. 'Tailored approach to sleep health education (TASHE): study protocol for a web-based randomized controlled trial', *Trials*, 17: 585.

**Wrong Intervention**

Corl, K., M. Levy, G. Phillips, K. Terry, M. Friedrich, and A. N. Trivedi. 2019. 'Racial And Ethnic Disparities In Care Following The New York State Sepsis Initiative', *Health Aff (Millwood)*, 38: 1119-26.

Parker, Margaret G., Mary-Lynn Drainoni, and Arvin Garg. 2023. 'Implementing a Social Determinants of Health Screening and Referral Care Model in the Neonatal Intensive Care Unit', Accessed 14 June 2024. <https://reporter.nih.gov/project-details/10365781#details>.
