# Supplementary material for: Interventions to improve racial and ethnic equity in critical care: A scoping review
Source: PLoS One. 2025 Nov 25;20(11):e0336922. doi: 10.1371/journal.pone.0336922 (PMC12646404; doi:10.1371/journal.pone.0336922)
Supplement: Attachment — Submitted filename: PLOS ONE Response to Reviewers_final.docx [file pone.0336922.s005.docx]

Emily Chenette, PhD

Editor-in-Chief

*PLOS One*

March 3, 2025,

Dear Dr. Chenette and the *PLOS One* Editorial Team,

Thank you for your consideration of our submitted manuscript “Interventions to improve racial and ethnic equity in critical care: A scoping review”. We are grateful for the reviewers’ thoughtful and constructive feedback. We have carefully considered each of their comments and have made the necessary revisions. Below, we provide a point-by-point response to the reviewers’ concerns. We believe these revisions have improved the rigor and impact of our manuscript. We hope that our revised manuscript now meets the standards for publication in PLOS One and look forward to your assessment.

*Reviewer #1: There are two limitations of this study that have not been discussed:*
*1) "We limited the scope of our review to the United States to enhance comparability of studies."*

*2) "Our scoping review did not seek to assess the quality of study designs because, as our review demonstrates, research to reduce disparities is at an early stage."*

*These two inclusion criteria could have limited external validity and increased the risk of bias. These two aspects should be highlighted in the discussion, and limitations regarding interpretation should be addressed.*

We have added both these limitations to the Discussion. We now also elaborate on the decision to limit our literature search to the United States because the U.S. has a distinct landscape of racial and ethnic disparities in healthcare shaped by its history, policies, and sociocultural dynamics (Lines 369-375).

*Even though inequity has been included as a search term, it has not been discussed, and references to use this perspective are missing.*

In Methods, we now describe how “inequity” is distinct from “inequality” or “disparity” and cite references for these distinctions. We then justify inclusion of “inequity” and “inequities” in our search strategy to capture all articles relevant to disparity research (Lines 124-126).

*Reviewer #2: Thank you for conducting this incredibly valuable work, and for the opportunity to review it for the team. My expertise lies in knowledge synthesis methodology generally and search methodology specifically, so that is where I will be focusing my feedback.
Data availability: this paper has good advice for what kind of data could/should be shared for knowledge syntheses. While you provide a lot, I think there's some "behind the curtain" stuff that could be shared, eg. templates, rough extraction data, list of full-text references that didn't meet inclusion, etc.
Page MJ, Nguyen PY, Hamilton DG, Haddaway NR, Kanukula R, Moher D, et al. Data and code availability statements in systematic reviews of interventions were often missing or inaccurate: a content analysis. J Clin Epidemiol [Internet]. 2022;147:1–10. Available from:* [*https://doi.org/10.1016/j.jclinepi.2022.03.003*](https://doi.org/10.1016/j.jclinepi.2022.03.003)*.*

We address this by adding new supplementary materials, including the PRISMA-S checklist and a list of the papers excluded during full-text review (see Supplementary Materials 1 and 3).

*Methods:
- the PRISMA you cite is for systematic reviews, please follow/cite the Scoping Review extension
- technically PRISMA is a reporting guideline only, not conduct. For conduct see JBI and Arksey & O'Malley.*

Thank you; we have clarified the citation of the PRISMA reporting guideline used. We have also clarified the language in the Methods section to clarify that we used the PRISMA-S guideline for reporting our procedures and results, not for guiding our procedures (Lines 91-94).

*- on page 4 you mention journals, conference abstracts and clinical trial registries, change subjects, and then report on the handsearching again - consider tightening this section up for clarity, and potentially making use of tables in the supplementary files (e.g. source searched, dates, keywords/method used to search, etc).*

We created Supplementary Material 2 which details the search strategy formatted to query each database, how many articles each database yielded, and further details on the search terms and yields of the hand search portion of our review. We also clarified the language in this paragraph (Lines 106-107).

*- does it make sense to report two database search approaches if ultimately you conducted the broader search anyway? I feel like this is eating up potential word count for results/discussion.*

We included both search strategies to highlight how we sought to specifically search for interventions targeting health disparities in critical care. As our study was a scoping review and we aimed to survey all available knowledge on our subject of interest, we conducted the broad database search last to ensure our search results were comprehensive.

*- (pg.6) PROSPERO is a systematic review protocol registry, does the team mean PRISMA?*

We indeed meant PRISMA-S. We replaced PROSPERO with PRISMA in the manuscript. Thank you for finding this error (Line 149).

*- please review PRISMA-S and report search strategies accordingly. They should be provided in full as run in the supplemental files. For example from how the searches are presented, it’s hard to know if subject headings were searched in addition to keywords, what fields were searched, etc.*

We now include the PRISMA-S checklist and all search strategies in the supplementary material (see Supplementary Material 1).

*- I'm a little concerned that the search may be overly simplistic (see for example the racial/ethnic search filters available here:* [*https://sites.google.com/a/york.ac.uk/issg-search-filters-resource/home/population-specific*](https://sites.google.com/a/york.ac.uk/issg-search-filters-resource/home/population-specific)*).*

We agree that this is a valid concern given the complexity of defining and capturing racial and ethnic disparities in healthcare research. However, our approach was guided by our specific research objective: to identify interventions addressing racial and ethnic disparities in critical care, *rather than disparities affecting a particular group*. Because of this broad scope, we designed a search strategy that was inclusive of all relevant studies, regardless of the population examined. To ensure methodological rigor, we collaborated with a research librarian, Hope Lappen, MLIS, MS, who has expertise in systematic and scoping review search strategies. Her input refined our search strategy, ensuring that it captured the breadth of relevant literature without being overly narrow or missing key studies.

*Results:
- I really like the way the team chose to break down results. It's very clear and the groupings are helpful for getting a bird's eye view.
- your tables are really clear and easy to read*

Thank you for these kind comments on our Results and Tables.

*Discussion:*
*- the Kilbourne phases is a helpful breakdown
- I think it's important here to emphasize that these are only American findings, and there may be different levels of development/interrogation of mechanism of action in other jurisdictions that are similar to the US (e.g. Canada, UK, Australia), or just globally, since every country has their own racial minorities. It would be helpful to contextualize your findings with the rest of the world, or if that's not feasible, frequently emphasize that these findings only relate to the US and not all of critical care literature.*

We now emphasize that these findings are specific to the US and discuss the rationale for and limitations inherent in this approach (Lines 369-375).

*- quality appraisal in scoping reviews are not common because they're intended to answer different questions. It might be simpler to just cite JBI here rather than getting into breadth of study designs.*

Thank you for this suggestion; we now cite JBI to support this point (Line 302-303, Reference #28).

*- I think your decision to limit to US-only is also a limitation, since NICUs/ICUs are pretty structured settings (it's not like we're comparing public health initiatives or something really nebulous) and could have had a lot to offer and provided richer context of where the literature is right now.*

We now address the US-only inclusion criterion as a limitation in the Discussion.

*Final thoughts: This was a really well written and thoughtful paper, and provides some very rich feedback for researchers in this field moving forward. I'm a bit concerned about the robustness of the search, especially if subject headings weren't used, as that is standard practice for knowledge synthesis searching. This would be easy enough to fix, though it would take this from a minor revisions to a major one since it would require additional screening, etc.*

We would like to thank the reviewer for their insights and suggestions. The subject headings of literature *were* included in the search; this is now evident in the supplementary material detailing all searches (Supplementary Material 2) and it is now mentioned in the Methods section (Lines 106-107).

We again sincerely appreciate the time and effort of the reviewers in providing thoughtful feedback on our manuscript. We hope that our revisions address the reviewers’ concerns and that our manuscript is now suitable for publication in PLOS One. Thank you for your consideration and we look forward to your response.

Sincerely,

Mari Armstrong-Hough, PhD, MPH

Associate Professor of Epidemiology and Social & Behavioral Sciences

New York University

+1 (212) 998-9015 | [mah842@nyu.edu](mailto:mah842@nyu.edu)

Shirley Ge, MD

Research Associate, AIRE Lab

New York University

+1 (978) 407-1314 | [shirleyge255@gmail.com](mailto:shirleyge255@gmail.com)
