# Supplementary material for: Interventions to improve racial and ethnic equity in critical care: A scoping review
Source: PLoS One. 2025 Nov 25;20(11):e0336922. doi: 10.1371/journal.pone.0336922 (PMC12646404; doi:10.1371/journal.pone.0336922)
Supplement: Attachment — Submitted filename: PLOS ONE Response to Reviewers_May.docx [file pone.0336922.s006.docx]

Emily Chenette, PhD

Editor-in-Chief

*PLOS One*

June 2, 2025

Dear Dr. Chenette and the *PLOS One* Editorial Team,

Thank you for your consideration of our revised manuscript “Interventions to improve racial and ethnic equity in critical care: A scoping review”. We are grateful for the reviewers’ thoughtful and constructive feedback. We have considered each of their comments and have made the necessary revisions. Below, we provide a point-by-point response to the reviewers’ concerns. We believe these revisions have improved the rigor and impact of our manuscript. We hope that our revised manuscript now meets the standards for publication in PLOS One and look forward to your assessment.

*Reviewer #2: Thank you very much for all the work you have put into revising this paper, already it is stronger!*

*Unfortunately, now that I can see the searches in full, they are not sufficiently robust to be published as part of a scoping review. The Web of Science search is fine as the database doesn't have subject headings, but PubMed, Embase and CINAHL all have subject headings that would directly apply to this topic (e.g. in PubMed: Ethnicity, Minority Groups, Race Factors, Racial Groups), and the use of both keywords and subject headings is recommended by all major synthesis bodies (Cochrane, JBI, etc). Some of these databases have mapping capabilites but they are not robust - when I tested the racial component of the search in PubMed and CINAHL the results were different with subject headings included. Here is an example of a broad race/ethnicity search for context (though it is for Ovid Medline): https://hsls.libguides.com/Ovid-Medline-search-filters/race-ethnicity#s-lg-box-32796073. The same goes for disparities, ICU, and to a lesser extent, quality improvement. All aspects of the search should have subject headings as well as keywords where applicable subject headings exist.*

*In addition to the fact that the search does not make use of subject headings, there are still numerous terms that I would expect to see in each category: minority group*, people/person/communit*/children/men/women of color/colour, equity, unequit*, unequal*, inadequat*, underserv*, disadvant*, depriv*, disproport*, bias, discriminat*, prejud*, racism, racist, potentially barrier*, critically ill, ECMO and ARDS acronyms spelled out, septic, potentially facilitat**

*I recommend the team revisit the searches with the librarian with an eye for subject headings and keywords (which you could limit to title/abstract only to keep numbers reasonable, as this is common practice for reviews), overlap this with what you've already screened and screen the difference.*

Co-author research librarian Hope Lappen revised and re-ran the search strategy to incorporate subject headings and additional keywords as suggested by the Reviewer. PubMed, Embase, and CINAHL were queried again with the new search strategy that included subject headings and the new keywords. Web of Science was also re-queried with the new search strategy to include the additional keywords. The Methods section is updated to reflect the new strategy (lines 101-128) and the revised search strategy for each database can be found in the updated Supplementary Material 2. An additional 813 studies were imported for screening, 39 duplicates were removed, resulting in an additional 774 studies screened. No additional studies meeting inclusion criteria were found (line 165). The PRISMA Flowchart (Figure 1) and the list of studies excluded in full-text review (S3) were updated accordingly.

*Reviewer #3: Very important and necessary contribution. I appreciated reading the in-depth description of how the team developed the taxonomy as well as how you decided to include or exclude studies (i.e. studies that had the 'wrong intervention').*

Thank you for this kind feedback on our study analysis.

*Please reframe the next steps (i.e. instead of calling for randomized control trials since it is unethical to perform this type of research in the social sciences by exposing a group to poor conditions for the purpose of investigation). Also, scoping reviews are not designed to assess the quality of research but the breadth so please update the statement that you conducted a rigorous evaluation of interventions aimed at eliminating disparities in critical care.*

For next steps, we suggested increased randomized control trials (RCTs, lines 314-322) because RCTs are generally the gold-standard study type to assess if an intervention is effective. Similar to the RCTs that met inclusion criteria in the scoping review, RCTs would expose study groups to a novel intervention aimed at reducing a pre-existing disparity in patient outcomes, instead of exposing a group to poor conditions to simulate a disparity. To illustrate, the RCT “Reducing Disparity in Receipt of Mother's Own Milk (ReDiMOM)” (Reference 34) randomized mother-infant pairs to either standard lactation care or an intervention designed to increase use of mother’s own milk and reduce disparities in breastfeeding rates between Black and non-Black infants. We highlight designs such as stepped-wedge cluster-randomized trials that allow all participating ICUs to benefit from the intervention under study (lines 318-320).

For assessing quality of research, we agree with the Reviewer as we discussed how we “did not seek to assess the quality of study designs because we aimed to survey current knowledge on reducing disparities in the ICU and, as our review demonstrates, research to reduce disparities is at an early state” (lines 317-318). In the Abstract, we did not aim to say that the scoping review was a rigorous evaluation of interventions, but rather that we only found a few interventions have been employed to address disparities in critical care. The Abstract has been edited to clarify this point (line 57).

*The abstract notes there were 86 studies that underwent full text review but the narrative lists that number as 75. Which was it?*

The narrative is consistent with the abstract as eighty-six studies underwent full-text review from which 75 studies were excluded (lines 50, 188, and 193 in the tracked changes manuscript). In the revised search in response to Reviewer #2, 93 studies underwent full-text review from which 82 studies were excluded (lines 49, 165, and 168).

*Since you cite the AMA's guidance on the need for the reviewed studies to define and frame race, will you elucidate the reason that your research team did not decide to also follow the AMA guidance on the reporting of race in your narrative (i.e. lowercase the 'w' in white):* [*https://www.ama-assn.org/system/files/ama-aamc-equity-guide.pdf*](https://www.ama-assn.org/system/files/ama-aamc-equity-guide.pdf)

*Otherwise, well done.*

The AMA-AAMC Equity Guide that is linked in the Reviewer’s comment is a different citation than the one cited in the manuscript, Updated Guidance on the Reporting of Race and Ethnicity in Medical and Science Journals. JAMA. 2021;326(7):621-7 (Reference 52). The Equity Guide to which the Reviewer refers is a communication handbook for clinical practice for when physicians are speaking and referring to patients and documenting care. Our cited reference informs the updated guidelines in reporting race and ethnicity for medical and scientific publications, as found in the eleventh edition of *AMA Manual of Style: A Guide for Authors and Editors*. We thus followed the *AMA Manual of Style* and capitalized the names of races including White.

We again sincerely appreciate the time and effort of the reviewers in providing thoughtful feedback on our manuscript. We hope that our revisions address the reviewers’ concerns and that our manuscript is now suitable for publication in PLOS One. Thank you for your consideration and we look forward to your response.

Sincerely,

Mari Armstrong-Hough, PhD, MPH

Associate Professor of Epidemiology and Social & Behavioral Sciences

New York University

+1 (212) 998-9015 | [mah842@nyu.edu](mailto:mah842@nyu.edu)

Shirley Ge, MD

Research Associate, AIRE Lab

New York University

+1 (978) 407-1314 | [shirleyge255@gmail.com](mailto:shirleyge255@gmail.com)
