# Supplementary material for: Interventions to improve racial and ethnic equity in critical care: A scoping review
Source: PLoS One. 2025 Nov 25;20(11):e0336922. doi: 10.1371/journal.pone.0336922 (PMC12646404; doi:10.1371/journal.pone.0336922)
Supplement: Attachment — Submitted filename: PLOS ONE Response to Reviewers_Aug_mah.docx [file pone.0336922.s007.docx]

Emily Chenette, PhD

Editor-in-Chief

*PLOS One*

August 11, 2025

Dear Dr. Chenette and the *PLOS One* Editorial Team,

Thank you for your consideration of our revised manuscript “Interventions to improve racial and ethnic equity in critical care: A scoping review”. We are grateful for the reviewers’ thoughtful and constructive feedback. We have considered each of their comments and have made the necessary revisions. Below, we provide a point-by-point response to the reviewers’ concerns. We believe these revisions have improved the rigor and impact of our manuscript. We hope that our revised manuscript now meets the standards for publication in PLOS One and look forward to your assessment.

*Reviewer #3: Great job with responding to the reviewer comments and sufficiently editing the manuscript .*

Thank you for this positive feedback.

*Reviewer#4: Dear Authors*

*Great work in submitting the revised version of the manuscript. A few comments to address,*

*The authors have suggested the need to randomized, controlled evaluations of interventions to promote equity in critical care – However, to design a well-controlled and adequately powered RCT the cost and resources, along with the sample size will be enormous. How about considering a SWAT (Studies within a trial) to identify racial and ethic differences in interventions (just a suggestion)*

We appreciate the reviewer’s suggestion to consider Studies Within a Trial (SWAT) as an alternative to large randomized controlled trials. While SWAT’s can identify subgroup differences within ongoing trials, we discussed how our results show that there is a paucity of intervention studies aiming to reduce racial or ethnic disparities in critical care despite there are already being many studies that detect racial or ethnic disparities in the ICU (Lines 269-291). Moreover, many of the studies meeting inclusion criteria focused on increasing advantageous health behaviors for all patients in practices that have been shown to have race or ethnic-based differences. These studies aimed to promote healthy behavior and then measured the impact on patients stratified by race, similar to the reviewer’s suggestion of SWAT’s (Lines 203-209).

*Line 67, references 7 to 9 – quotes one of the author’s earlier papers, I am wondering if this will that be a potential conflict of interest for this scoping review?*

We do not feel citing a co-author’s previous paper, *Ethnic Disparities in Deep Sedation of Patients with Acute Respiratory Distress Syndrome in the United States: Secondary Analysis of a Multicenter Randomized Trial* (reference 9), is a conflict of interest because we cite the paper to provide background on the evidence for ethnic disparities in ICU care in addition to several other examples from other papers by different authors (Lines 64-70, references 1-15). This paper is not included in the scoping review itself, nor would it be eligible because it does not report or evaluate an intervention.

*Line 84, 85 – “to consolidate the available knowledge regarding interventions and quality improvement initiatives to reduce racial and ethnic disparities in critical care.”
I am not sure how much of the second objective has been satisfied with this review, it might be helpful to highlight this one.*

We only had one primary objective to consolidate the current knowledge on interventions and quality improvement initiatives to reduce racial ethnic disparities in critical care by reviewing existing and ongoing studies (Lines 82-85). Table 1 summarizes our findings of the current knowledge and we comment on the implications of our results in the Discussion (Lines 268-351).

*The authors have attempted to address the racial and ethnic disparities in the studies – it might be helpful to add how much was the ethnic variability in the study team, might be interesting from a reader perspective to know about the team (please ignore this if you think it’s inappropriate)*

Our objective was to synthesize evidence on interventions to address racial and ethnic disparities in critical care, and we believe the focus should remain on the content and quality of the included studies rather than on the demographics of the review team.

*I am not sure if PRISMA standard or PRISMA ScR checklist is suitable for use in scoping reviews?*

Thank you for this feedback. We have replaced the PRIMSA standard checklist with the PRISMA extension for scoping reviews (PRISMA-ScR) as PRISMA-ScR is specifically for scoping reviews (Supplementary Material 1).

*The authors have aimed to addressed the disparities in US – just curious to know why, a few statements to strengthen why studying about US might be helpful.*

Non-U.S. studies were excluded because racial and ethnic disparities in healthcare in the United States arise from a distinct combination of historical, policy, and sociocultural factors. Studies conducted in other countries reflect disparities shaped by their own sociopolitical environments and healthcare systems, which limits the applicability and comparability of their findings to the U.S. context (Lines 361-365).

*In the results section – it might be helpful to mention the race and ethnicity of the population studied in the included studies.
It might be helpful to highlight the ongoing studies in table 1 with an easy identifier (different color or *) for easy identification.
Table 1 – it might be helpful to add the total number of sample size along with the % of race and ethnicity within the studies.
Table 2 is something I really liked. Well done.
It might be better to add the strengths of the study before the limitations.*

Thank you to the reviewer for these suggestions. To table 1, we added indicators for ongoing clinical trials and studies with interventions that continued to be implemented past the study period with “*” and “†”, respectively. Table 1 already includes a column for study population. Regarding the sample size and proportion of race and ethnicity within each study population, the specific demographics of participants, beyond the targeted population already included in the table, are not relevant to answering the study objective. We sought to consolidate the current knowledge on interventions and quality improvement initiatives to reduce racial ethnic disparities in critical care by reviewing existing and ongoing studies in a scoping review (Lines 82-85). Unlike a systematic review, demographics and outcomes of included studies are not always necessary to answer the research question.

We again sincerely appreciate the time and effort of the reviewers in providing thoughtful feedback on our manuscript. We hope that our revisions address the reviewers’ concerns and that our manuscript is now suitable for publication in PLOS One. Thank you for your consideration and we look forward to your response.

Sincerely,

Mari Armstrong-Hough, PhD, MPH

Associate Professor of Epidemiology and Social & Behavioral Sciences

New York University

+1 (212) 998-9015 | [mah842@nyu.edu](mailto:mah842@nyu.edu)

Shirley Ge, MD

Research Associate, AIRE Lab

New York University

+1 (978) 407-1314 | [shirleyge255@gmail.com](mailto:shirleyge255@gmail.com)
