# Supplementary material for: Interventions to improve racial and ethnic equity in critical care: A scoping review
Source: PLoS One. 2025 Nov 25;20(11):e0336922. doi: 10.1371/journal.pone.0336922 (PMC12646404; doi:10.1371/journal.pone.0336922)
Supplement: Attachment — Submitted filename: PLOS ONE Response to Reviewers_Oct.docx [file pone.0336922.s008.docx]

Emily Chenette, PhD

Editor-in-Chief

*PLOS One*

October 7, 2025

Dear Dr. Chenette and the *PLOS One* Editorial Team,

Thank you for your consideration of our revised manuscript “Interventions to improve racial and ethnic equity in critical care: A scoping review”. We are grateful for the reviewers’ thoughtful and constructive feedback. We have considered each of their comments and have made the necessary revisions. Below, we provide a point-by-point response to the reviewers’ concerns. We believe these revisions have improved the rigor and impact of our manuscript. We hope that our revised manuscript now meets the standards for publication in PLOS One and look forward to your assessment.

*Reviewer #3: Great job responding in detail to all of the reviewer comments thus far. One last question I have is why your team did not elect to rely on 3 researchers for the screening/review of studies (2 initial reviewers and 1 to break ties). This is the standard way to pursue interrater reliability. Please add a line as to why you opted to rely on 2 researchers rather than 3.*

Research librarian Hope Lappen was often consulted as the third reviewer on studies for which the initial two reviewers had difficulty reaching a consensus. A consensus was reached with Lappen’s expertise or with Lappen casting a tie-breaking vote. Her role as the third reviewer was added to the Methods section (Line 139-140).

*Reviewer #4: Dear Authors,*

*The submitted manuscript reads very well.*

*Please modify the PRISMA to PRISMA ScR (more than two places still read as PRISMA). In the discussion, I will encourage you to add the strengths of the study before the limitations (content from line 366-371 may be placed before line 352).*

*All the best!*

PRISMA was modified to PRISMA-ScR in the manuscript text and in the title of Figure 1. The discussion of the study strengths was moved before the limitations.

We again sincerely appreciate the time and effort of the reviewers in providing thoughtful feedback on our manuscript. We hope that our revisions address the reviewers’ concerns and that our manuscript is now suitable for publication in PLOS One. Thank you for your consideration and we look forward to your response.

Sincerely,

Mari Armstrong-Hough, PhD, MPH

Associate Professor of Epidemiology and Social & Behavioral Sciences

New York University

+1 (212) 998-9015 | [mah842@nyu.edu](mailto:mah842@nyu.edu)

Shirley Ge, MD

Research Associate, AIRE Lab

New York University

+1 (978) 407-1314 | [shirleyge255@gmail.com](mailto:shirleyge255@gmail.com)
